# Can self-representationalism explain away the apparent irreducibility of consciousness?

**DOI:** 10.1007/s11229-015-0806-1

**Published:** 2015-07-04

**Authors:** Tom McClelland

**Affiliations:** School of Social Sciences, University of Manchester, Oxford Road, Manchester, M13 9PL UK

**Keywords:** Consciousness, The Hard Problem, Self-representationalism, Reduction, Qualitative character, Subjectivity, Russellian physicalism

## Abstract

Kriegel’s self-representationalist (SR) theory of phenomenal consciousness pursues two projects. The first is to offer a positive account of how conscious experience arises from physical brain processes. The second is to explain why consciousness misleadingly appears to be irreducible to the physical i.e. to ‘demystify’ consciousness. This paper seeks to determine whether SR succeeds on the second project. Kriegel trades on a distinction between the subjective character and qualitative character of conscious states. Subjective character is the property of being a conscious state at all, while qualitative character determines what it is like to be in that state. Kriegel claims that SR explains why subjective character misleadingly appears irreducible, thereby neutralising the apparent irreducibility of consciousness. I argue that although SR credibly demystifies subjective character, it cannot explain why qualitative character also appears irreducible. I conclude that we should pursue the possibility of a hybrid position that combines SR with an account that does explain the apparent irreducibility of qualitative character.

## Introduction

Kriegel’s self-representationalist (SR) theory of consciousness does not just put forward an explanation of consciousness amenable to physicalism, it also attempts to explain away the intuition that consciousness is physically irreducible. Although Kriegel’s proposed account of consciousness has received substantial attention in recent years, his distinctive account of the source of anti-physicalist intuitions has gone relatively unnoticed. The purpose of this paper is to determine whether SR can indeed explain why consciousness misleadingly appears irreducible. As such I will not be assessing Kriegel’s claim that SR is true, but rather his conditional claim that *if *SR is true *then* the apparent irreducibility of consciousness can be explained away. In Sect. 3 I reconstruct Kriegel’s proposal and in Sect. 4 I assess its value. I will argue that although SR does provide a *partial* explanation of the apparent irreducibility of consciousness, it does not provide the *complete* explanation that Kriegel promises. Specifically, I suggest that Kriegel credibly explains why subjective character—the property of there being *something it’s like* to be in a conscious state—appears irreducible. He does not, however, explain why qualitative character—the properties that constitute *what *it’s like to be in a conscious state—also appears irreducible. I argue that Kriegel’s proposal ought to be combined with an account that can explain away the apparent irreducibility of qualitative character, and propose that Russellian physicalism offers just such an account. Before we get to the main argument of the paper though, Sect. 2 sets up the terms of the debate and pins down the crucial distinction between explaining consciousness and explaining its apparent irreducibility.

## The apparent irreducibility of consciousness

### Phenomenal consciousness

Kriegel follows Levine (2003, pp. 6–7) in distinguishing between two aspects of phenomenally conscious mental states: their *subjective character* and their *qualitative character*. A mental state has subjective character iff there is *something it’s like to be* in that state for the subject of that state. It is subjective character that differentiates conscious from non-conscious mental states: a conscious mental state is one that exists *for the subject* who is in that state while non-conscious mental states are those that do not. A mental state becomes conscious precisely when it becomes subjectively presented, and sinks back into unconsciousness precisely when it loses that feature. A subject is phenomenally conscious at all in virtue of being in a subjective state.^1^

While subjective character is what differentiates conscious from non-conscious mental states, qualitative character is what differentiates types of conscious state from one another. The qualitative character of a conscious state is *what it’s like to be* in that state for its subject. Your current experience will be characterised by a vast array of phenomenal qualities: the black quality of the words before you, the white quality of the background, perhaps an itchy quality in your foot and a dull ache in your shoulders. These qualities are ways in which experiences resemble and differ from each other phenomenologically. As Kriegel explains, ‘...the qualitative character of a conscious experience at a time is given by the sum of qualitative properties it instantiates at a time’ (2009, p. 46). A mental state is a phenomenal state at all in virtue of having subjective character, but is the kind of phenomenal state it is in virtue of its qualitative character.

One account of the relationship between subjective and qualitative character is that they stand in something like a determinable/determinate relation. On this view, no state is conscious (i.e. has subjective character) without being conscious in some particular way (i.e. having some specific qualitative character), and no state can have qualitative character without thereby having subjective character. Drawing on Kriegel’s terminology we can call this the ‘inseparatist’ model (2009, p. 53). An alternative model is ‘separatism’ according to which the subjective and qualitative character of a conscious state are discrete properties that can be instantiated independently. On this view, a state could have qualitative without subjective character or subjective without qualitative character. Our phenomenal states would involve a conjunction of both properties.^2^ Adjudicating on which model is most credible is a complex issue beyond the scope of this paper. I raise this issue only to emphasise that the distinction between subjective and qualitative is *neutral *on which metaphysical model is accurate.

### The appearance of irreducibility

Physicalism is true iff phenomenal states are nothing more than physical states. This means that neither the subjective nor qualitative character of conscious states can involve the instantiation of non-physical properties. It should be uncontroversial to place the following metaphysical condition on physicalism: *physicalism is true only if the physical facts necessitate the phenomenal facts.* It is much more controversial to hold that this metaphysical condition brings with it the following epistemic commitment: *the necessitation of the phenomenal facts by the physical facts must be epistemically transparent.* There is, of course, an extensive debate surrounding this epistemic commitment: one side says that physicalism must satisfy this condition while the other side says it does not. Rather than getting side-tracked by this familiar debate, I will stipulate that physicalism must indeed satisfy the epistemic condition. My justification for this is threefold: (a) I believe that a very strong case can ultimately be made in favour of the epistemic condition; (b) Kriegel himself offers a convincing case for the epistemic condition (2009, pp. 273–283); (c) Kriegel is targeting anti-physicalists who presuppose the epistemic condition, meaning that it should be granted for dialectical purposes.

It appears, to many at least, that consciousness is irreducible to physical goings-on. More precisely, it appears that there can be no epistemically transparent entailment from the physical facts to the phenomenal facts. The thought here is not just that some specific physicalist theory fails to explain consciousness. Rather, the thought is that phenomenal facts are simply the *wrong kind* of fact to be entailed by physical facts. No matter what physical facts you cite, the phenomenal facts will not be entailed: it will inevitably remain a *further question* how things stand phenomenally. The appearance of irreducibility can be most vividly highlighted using conceivability intuitions. If the phenomenal facts reduce to the physical facts, then it is epistemically impossible for the physical facts to hold but for the phenomenal facts to be other than they actually are. And if a scenario is epistemically impossible it should be inconceivable. Yet it appears that given any set of physical facts about the actual world, one can conceive of the phenomenal facts being other than they actually are.

The distinction between subjective and qualitative character provides us with a useful distinction between two different ways in which we might conceive of the phenomenal facts varying. First, one can conceive of the physical facts being as they are and yet facts about the distribution of subjective character being different. This comes out in the ‘zombie’ scenario: we can apparently conceive of a being like ourselves in all physical respects but who is such that there is *nothing it’s like to be *that subject (Chalmers 1996, p. 94). It thus appears that the subjective character of conscious states is irreducible to physical goings-on. Second, one can conceive of the physical facts being as they are and yet the facts about qualitative character being different. This comes out in the ‘qualia-inversion’ scenario: we can apparently conceive of a being like ourselves in all physical respects but who is such that *what it’s like to be *that subject is different to what it’s like to be us. Specifically, the colour-qualities that characterise our twin’s visual experience are inverted relative to our own (Shoemaker 1982). It thus seems that the qualitative character of conscious states is irreducible to physical goings-on. If these scenarios are indeed conceivable, then the physical facts do not epistemically entail the phenomenal facts.^3^

### Demystifying consciousness

Why does consciousness appear irreducible? Anti-physicalists say that it appears irreducible because it *really is* irreducible. Although this might be sound as an explanation of the appearance, a number of well-established problems make anti-physicalism a very unattractive position.^4^ Physicalism offers a far more credible metaphysical picture, but must maintain that the apparent irreducibility of consciousness is deceptive. To ‘demystify’ consciousness is to explain away its apparent irreducibility. This means acknowledging that consciousness *seems *non-physical whilst offering an account of this appearance that doesn’t require consciousness to *actuallybe* non-physical. Demystifying consciousness need not even involve giving any positive reasons to believe that physicalism is true. Instead, it is to do with neutralising a compelling reason to doubt that physicalism is true. It is important to recognise that the project of demystifying consciousness is quite different to the project of proposing an explanation of consciousness amenable to physicalism. One way to make this distinction vivid is to consider theories that pursue one of these projects but not the other.

Baars (1988) wields a range of experimental evidence to support his global workspace theory (GWT) according to which a mental state is conscious in virtue of being broadcast to a large distributed set of receiving assemblies. Although GWT has a great deal to say about the physical basis of conscious experience, it has nothing to say about intuitions of irreducibility (see Chalmers 1996, p. 112). Faced with an anti-physicalist who insists that they can conceive of these cognitive processes going on in the absence of consciousness, Baars will have to defer to someone who addresses intuitions of irreducibility directly. Even if GWF is true, further work would need to be done to demystify consciousness.

The phenomenal concept strategy holds that the concepts we use to think about our conscious states are cognitively isolated from our concepts of physical properties (see Stoljar 2005). This allegedly generates the appearance that our phenomenal concepts do not designate physical properties, and enables us to conceive of phenomenal properties varying independently of their physical basis. This kind of account tells a detailed story about why consciousness appears irreducible, but will have nothing to say about the actual physical basis of conscious experience. In fact, it is even compatible with consciousness being non-physical. The aim of the phenomenal concept strategy is not to explain consciousness but to demystify it. Faced with a scientist wanting to know how consciousness arises from physical processes, the advocate of this view will have to defer to our leading theories of consciousness. Even if the phenomenal concept strategy is successful, further work would be needed to give a positive physicalist theory of consciousness.

I am unconvinced that either GWT or the phenomenal concept strategy succeed in their respective projects. My point, though, is that their respective projects are clearly different. The interesting thing about Kriegel’s self-representationalism is that it promises to succeed on *both* projects at once. In the first seven chapters of his (2009), Kriegel develops his representational theory of how consciousness arises from physical processes. He recognises, however, that like any physicalist theory of consciousness SR leaves us with an unresolved sense of mystery—a residual intuition that the proposed explanation of consciousness is incomplete. It is this appearance of irreducibility that he seeks to demystify in his eighth and final chapter. We can now move on to examine exactly how this proposed demystification proceeds.

## The self-representationalist proposal

Kriegel argues: (a) that SR is true and; (b) that if SR is true then the apparent irreducibility of consciousness can be explained away. Although I am concerned only with the second of these claims, in Sect. 3.1 I will outline Kriegel’s case for the first thesis. This will serve to elucidate Sr’s account of consciousness, setting us up to explore the second claim in Sect. 3.2.

### The self-representationalist theory of consciousness

Kriegel’s view is that ‘...phenomenally conscious states have qualitative character in virtue of representing environmental features and subjective character in virtue of representing *themselves*’ (2009, p. 2). The bulk of Kriegel’s argument concerns the second of these claims. He seeks to show that ‘...what makes something a conscious state at all, what constitutes its subjective character, is a certain kind of *self-representation*...’ (2009, p. 13). Kriegel offers the following ‘master argument’ for the thesis that any conscious state C is conscious in virtue of suitably representing itself:

1. C is conscious in virtue of being suitably represented;
2. it is not the case that C is conscious in virtue of being represented by a numerically distinct state; therefore,
3. C is conscious in virtue of being suitably represented by itself...(2009, pp. 15–16)

The thinking behind premise 1 is that a mental state is a *conscious *mental state when we are appropriately *aware *of it. Since awareness is plausibly a representational notion, we can conclude that our conscious mental states are conscious in virtue of being suitably represented by us. This simple insight motivates the higher-order representation (HOR) theories of consciousness (e.g. Rosenthal 1986; Lycan 2001). On such views, C is conscious in virtue of being suitably represented by *another* of the subject’s mental states. In a sense, it is the *failings* of HOR theories that justify premise 2. Regarding the HOR as distinct from its object leads to a number of problems. By claiming that the higher-order content that makes a state conscious must belong to that very state, SR credibly overcomes these problems. These problems are well known so I will only sketch them here.

First, HOR theories are committed to the possibility of targetless HORs. When asked whether such representations suffice for consciousness the HOR theorist must make an apparently arbitrary choice between two answers, each of which entails difficulties. SR, by contrast, entails that targetless HORs (of the relevant kind) are impossible. If a mental state represents *itself* it cannot fail to have a target, so the dilemma is avoided (Kriegel 2009, pp. 129–139). Second, by saying that a state is conscious in virtue of its *relational* property of being represented by some distinct mental state, the HOR theorist has no principled reason to deny that *non-mental *states that stand in this relation are also conscious. This ‘generality problem’ is avoided by SR since only mental states can (suitably) represent themselves (Kriegel 2009, p. 145). Third, upon phenomenological reflection many claim that their awareness, and that of which they are aware, do not appear to be distinct states. Unlike HOR theories, SR can accommodate this datum straightforwardly: they do not seem like distinct states because they *really aren’t* distinct states (Kriegel 2009, pp. 146–155).

Returning to the master argument, it is clear that the conclusion that SR is true follows from the two premises. Kriegel goes on to flesh out the ‘suitable’ self-representation clause in a way that eliminates some obvious potential counter-examples (2009, pp. 157–164). This eventually leads us to the following formulation of the self-representational account of subjective character:

Necessarily, for any mental state M, M has subjective character iff M is non-derivatively, specifically, and essentially self-representing. (Kriegel 2009, p. 164)

How does a conscious state achieve this self-representation? On Kriegel’s account, a conscious state is a complex state with two components: a lower-order component $\hbox {M}_{1}$ that represents the world and a higher-order component $\hbox {M}_{2}$ that represents the lower-order component. Kriegel suggests that we often represent entities *indirectly* in virtue of directly representing a part of that entity. In the case of a conscious state, $\hbox {M}_{1}$ directly represents $\hbox {M}_{2}$ and thereby indirectly represents the whole conscious state of which both are parts (Kriegel 2009, p. 220).

Kriegel’s full theory of phenomenal consciousness also includes an account of qualitative character. Although I will examine this account in Sect. 4, I put it aside presently as it is specifically Kriegel’s self-representational account of subjective character that drives his demystification of consciousness. His account of qualitative character plays no role in his account of why consciousness appears irreducible.

### SR’s demystification of consciousness

Kriegel promises to explain why consciousness appears irreducible, but before embarking on this project he focuses the scope of his proposed explanation. He ties the apparent irreducibility of conscious states specifically to the apparent irreducibility of their *subjective character* i.e. to their property of being conscious states at all. He notes that it appears to be an open question whether a mental state is conscious even when all the physical facts are set (2009, p. 272), and notes the apparent conceivability of zombie duplicates (2009, p. 284). The reducibility of qualitative character—of the properties that determine what a conscious state is like—is not considered by Kriegel here. In the next section I will argue that this crucial move is mistaken, but for now I will focus on whether Kriegel can achieve his stated goal of demystifying subjective character.

What does it mean for subjective character to appear irreducible? It cannot just mean that existing attempts to reduce subjective character appear to fail. That would be a case of a property appearing *unreduced*, not of it appearing *irreducible*. For subjective character to seem irreducible is for it to seem like the *wrong kind *of property to ever be given a physical reduction. Kriegel fleshes this out with the suggestion that subjective character appears non-functionalizable so, on the assumption that non-functionalizability precludes reduction, it would thus appear irreducible. Here Kriegel follows Chalmers (1996), Levine (2003) and others in equating the apparent irreducibility of subjective character with its apparent non-functionalizability. I recommend using the distinction between 1st- and 2nd-order properties to illuminate this view. Having subjective character is a 1st-order property of all and only conscious states. Being non-functionalizable is a 2nd-order property. The suggestion is: (a) that subjective character appears to have this 2nd-order property and; (b) that having this 2nd-order property precludes a 1st-order property from being reduced to the physical. This is what the apparent irreducibility of subjective character consists in, so this is the appearance that Kriegel ultimately needs to explain. Overall, the case against physicalism that Kriegel implicitly attributes to his opponent can be reconstructed as follows:

1. A property is reducible only if it is functionalizable.
2. Therefore subjective character is reducible only if it is functionalizable.
3. Subjective character uniquely appears non-functionalizable.
4. Subjective character uniquely appears non-functionalizable because it *really is* uniquely non-functionalizable.
5. Therefore consciousness is irreducible.

Premise 1 is motivated by a general model of reduction most forcibly presented by Chalmers and Jackson (2001). Take the familiar case of the reduction of water to $\hbox {H}_{2}\hbox {O}$. On this model, knowledge of this reduction comes in two stages. In the first stage the subject needs a proper understanding of the meaning of ‘water’. Chalmers and Jackson offer a functional account of the meaning of ‘water’ whereby water is whatever performs the *functional role* definitive of being water. A thing’s functional role is ‘...given by its bundle of dispositions to cause and be caused by certain things’ (Kriegel 2009, p. 275 ft. 11). Thus, to be water is to be the *X* such that it is potable, transparent, quenches thirst, falls from the sky etc. A property is functionalizable just in case its nature is exhausted by some functional role. Although Chalmers and Jackson hold that we can establish what the ‘water role’ is a priori other models acknowledge that functional roles may sometimes need to be discovered a posteriori (Kriegel 2009, p. 285). In the second stage, the subject learns that the substance $\hbox {H}_{2}\hbox {O}$ is what performs the water role in the actual world. This is the a posteriori insight that $\hbox {H}_{2}\hbox {O}$ is the actual stuff that is potable, transparent etc. From this, the subject can then deduce that water is $\hbox {H}_{2}\hbox {O}$. Once you fix the functional analysis of water and fix the facts about what $\hbox {H}_{2}\hbox {O}$ does, it is not an open question whether or not $\hbox {H}_{2}\hbox {O}$ is water.

It is credible (though controversial) that *all *reduction follows this general model.^5^ Reduction involves showing that the instantiation of the reduced property requires nothing more than the performance of some causal role, and that the performance of that causal role requires nothing more than the instantiation of the properties in the reduction base. Applying this to the case of subjective character, we get premise 2. To reduce subjective character would involve *functionalizing* it and then showing that the relevant functional role is performed by physical properties. It is the first of these steps that is problematic, hence premise 3. It seems that there is more to subjective character than the performance of some causal role. Even if a physical state occupies the causal role distinctive to mental states with subjective character, it would remain a further question whether that physical state is accompanied by consciousness. This is because subjective character is a non-functionalizable property—its functional role (whatever that might be) does not exhaust its nature.

This captures how the apparent *non-functionalizability* of consciousness could be responsible for its apparent *irreducibility*. But *why* does consciousness appear non-functionalizable? To explain this would be to explain our intuition of irreducibility. Of course, anti-physicalists say that it appears non-functionalizable because it *is* non-functionalizable. But this is where the physicalist should be trying to block the argument for irreducibility. *Yes*, consciousness uniquely *appears* non-functionalizable but *No*, that appearance need not reflect consciousness *really being* non-functionalizable.

In order to understand why subjective character seems non-functionalizable it is worth reflecting on why other properties (perhaps *all* other concrete properties) appear open to functionalization. Kriegel has a plausible story to tell about this. We gain epistemic access to properties in virtue of their causal impact upon us. Sometimes this causal impact is relatively immediate, as in perception. Other times it is more circuitous, as in the detection of properties using sophisticated scientific instruments. Because we can *only* know a property through how it affects us, it is inevitable that the criteria we have for that property being instantiated will be exclusively causal. For instance, our criteria for *being water* will involve nothing more than *doing what water does*. After all, if there were anything more than this to being water we would have no way of gaining epistemic access to it.

If normal properties appear functionalizable because they are known exclusively via their causal role, perhaps subjective character appears *non-*functionalizable because it is *not* known exclusively via its causal role. SR has interesting implications for our epistemic access to subjective character and Kriegel argues that this distinctive epistemic status is responsible for the appearance that consciousness is non-functionalizable. Because conscious states are self-representing, our epistemic access to the subjective character of our conscious state is built into the very instantiation of consciousness. A subject’s knowledge of subjective character is not mediated by some causal chain running from an instantiation of consciousness to the subject. Rather, a subject gains causally unmediated acquaintance with subjective character simply by *being in a conscious state*. This is not to say that consciousness guarantees the presence of a *belief* that one is conscious—such propositional knowledge plausibly requires a further cognitive step. Rather, the idea is that when we are in a state of awareness, we are thereby *aware of our awareness*. By instantiating the property of being conscious, a subject is put into epistemic contact with that very property. As Kriegel puts it, ‘...knowledge of consciousness, and of consciousness alone, does not require causal contact with the known’ (2009, p. 295). Crucially though, even though conscious states yield this special non-causal kind of knowledge they are still, according to Kriegel, wholly analysable in causal terms.

Kriegel’s suggestion is that the non-causal access we have to subjective character is what generates our intuition that it is a property that transcends any purely causal characterisation i.e. a non-functional property. This appearance, however, is not to be trusted. Assume, for the sake of argument, that the instantiation of subjective character is nothing more than the instantiation of some functional property F: the instantiation of F suffices for suitable self-representation, which in turn suffices for being in a state with subjective character. If this were the case, SR predicts that we would have a dual epistemic access to F. We would know F *from the outside* via its outward causal manifestations. We would also know F *from the inside* by ourselves instantiating it. This would create the appearance that the property to which we have that special inside access is *something over and above* the functional property we can access from the outside. After all, when we discern that the conscious state we are in has subjective character, we don’t need to tick off a check-list of causal properties possessed by that state. Instead, the subjective character of our conscious state reveals itself to us directly.

So Kriegel’s core claim is that the fact that we have a way of accessing our own consciousness other than via its functional role does not show that something over and above the performance of the right functional role is required for consciousness to be instantiated. Put another way, the fact that our *criteria* for subjective character being instantiated are non-functional does not show that *subjective character itself* is non-functional. Physicalists are thus free to tell the following functional story about consciousness: the occurrence of physical properties performing a certain causal role suffices for the instantiation of F; F suffices for the instantiation of a suitably self-representing mental state; such a representational state suffices for the instantiation of a conscious state; and finally, the occurrence of a conscious state suffices for the subject of that state having non-causal knowledge of their own consciousness. This knowledge is non-causal in that the subject has epistemic access to their conscious state simply by being in it, and not by standing in some causal relation to it. That said, it is the causal properties of conscious states and their parts that make such non-causal knowledge possible. In other words, the non-causal epistemology of consciousness can be given a purely causal metaphysics.

Kriegel’s account of the mechanism of self-representation provides us with some details about the functional underpinnings of consciousness: a conscious state is a complex of two mental states $\hbox {M}_{1}$ and $\hbox {M}_{2}$ such that: (a) $\hbox {M}_{2}$ stands in a causal relation to $\hbox {M}_{1}$ in virtue of which it directly represents $\hbox {M}_{1}$ (2009, pp. 224–228) and; (b) the neural realisers of $\hbox {M}_{1}$ and $\hbox {M}_{2}$ stand in a relation of neural synchrony in virtue of which both states are *integrated *into a complex whole, where neural synchrony consists in neural states sharing the functionalizable property of firing at a particular rate (2009, pp. 245–248).^6^ Here self-representation is given an exclusively functional analysis. The fact that the subject’s experience does not disclose its functional underpinnings does not show that those underpinnings are not present, nor does it show that it is possible for the experience to exist in the absence of those underpinnings.

None of this confirms that consciousness is in fact a functional property. It does, however, undermine our central reason for doubting that this is so. The apparent non-functionalizability of consciousness should not be taken at face value as it would still have this appearance even if it was in fact a functional property.^7^ It is permissible for physicalists to posit that consciousness *is* in fact a functional property but that, due to the epistemic considerations uncovered by SR, it is a functional property that misleadingly seems non-functionalizable to subjects who instantiate it. Consciousness does indeed appear non-functionalizable, and thus irreducible. But the reason for this ‘...is not that consciousness can *occur *independently of its functional profile, but that it can be *known* independently of its functional profile’ (Kriegel 2009, p. 299).

## Does SR demystify consciousness?

Kriegel claims that SR demystifies consciousness. Implicit in his proposed demystification is the following argument:

1. SR demystifies subjective character.
2. If SR demystifies subjective character, then SR demystifies consciousness.
3. Therefore, SR demystifies consciousness.

The discussion in the previous section suggests that premise 1 is highly plausible: SR predicts that subjective character appears irreducible but also shows why that appearance is not to be trusted. My objection to Kriegel concerns his (somewhat suppressed) premise 2. I claim that the qualitative character of conscious states also appears irreducible, and that SR does nothing to neutralise this appearance of irreducibility. In Sect. 4.1 I argue that the demystification of consciousness requires the demystification of qualitative character. In Sect. 4.2 I argue that SR is not equipped to demystify qualitative character. In Sect. 4.3 I argue that my objections to Kriegel’s proposal do not entail that it should be dismissed entirely. Instead, we should pursue the possibility of a hybrid account that combines SR’s demystification of subjective character with some alternative demystification of qualitative character.

### Does SR owe us a demystification of qualitative character?

The phenomenal qualities of our conscious states appear to be inexplicable in physical terms. Consequently, even if the subjective character of consciousness could be demystified, we would still be left with something about consciousness that seems to resist reduction. This claim breaks down into two theses: (a) that qualitative character appears irreducible and; (b) that the demystification of consciousness therefore requires the demystification of qualitative character. There are thus two possible ways in which Kriegel might resist my claim: he can deny that qualitative character appears irreducible or he can accept its apparent irreducibility but deny its relevance to the demystification of consciousness. I argue that neither route is feasible, and that SR therefore owes us a demystification of qualitative character.

In line with the first route, Kriegel comes quite close to denying that qualitative character appears to resist reduction. He writes:

...although the deep mystery of consciousness does have to do with why and how conscious episodes differ from each other, it is much more concerned with why and how there are conscious episodes to begin with—that is, why there exists such a thing as consciousness at all. (2009, p. 11)

The suggestion here is not that there is no need to explain qualitative character. Kriegel’s theory does, after all, include a proposed explanation of qualitative character. Instead, the suggestion is that qualitative character does not appear inexplicable in physical terms (or at least that it doesn’t appear *as *inexplicable as subjective character). If there is nothing about phenomenal qualities that makes them compellingly appear to be properties that resist reduction, then there is nothing about qualitative character that stands in need of demystification.

My initial response to this defence is an appeal to intuition: isn’t it just the case that qualitative character *does* appear inexplicable in physical terms? Consider, for instance, the claim that the phenomenal quality of pain is nothing more than the firing of C-fibers. Many agree with Levine’s suggestion that:

...what is left unexplained by the discovery of C-fiber firing is *why pain should feel the way it does! *For there seems to be nothing about C-fiber firing which makes it naturally “fit” the phenomenal properties of pain, any more than it would fit some other set of phenomenal properties. (Levine 2003, p. 356)

The sense of mystery also comes out vividly in qualia-inversion cases: it appears conceivable that two subjects could be alike in all physical respects and yet differ with respect to the qualitative character of their respective experiences. Against this, Kriegel can insist that there’s no real problem here—that there’s nothing mysterious about why physically-realised conscious states have the qualitative character they have. This would, however, put him in an uncomfortable dialectical position. Kriegel does take seriously the intuition that zombies are conceivable: indeed, it underwrites his case for regarding subjective character as mysterious. The intuition that qualia-inverts are conceivable seems to have parity with the first intuition. Consequently it looks inconsistent, or at least ad hoc, for Kriegel not to take the second intuition equally seriously. If conceivability intuitions are taken seriously regarding zombies, they should be taken equally seriously regarding qualia-inverts. At the very least, good reasons need to be given for doubting that the two cases have parity, yet Kriegel provides no such reasons. Put another way, if Kriegel denies that qualia-inversion intuitions indicate that qualitative character is mysterious, he cannot comfortably maintain that zombie intuitions indicate that subjective character is mysterious.

Although Kriegel cannot credibly deny that qualitative character appears irreducible, he might be able to deny that this is his problem. SR promises to demystify consciousness. If the mystery of qualitative character is not a mystery that strictly pertains to consciousness, then SR does not owe us an account of it.^8^ One promising view of qualitative character is that phenomenal qualities are external properties that are represented by our conscious states. An experience with a reddish quality, for instance, is an experience that suitably represents the property of being red. Such external properties raise deep metaphysical problems. The redness of a ripe tomato, for instance, appears irreducible to its physical properties. No matter what underlying physical properties we cite, it remains a mystery why those properties would be associated with a red quality rather than, say, a green quality. Although there is a sense in which the reddishness of our experience seems mysterious, Kriegel suggests ‘...the source of the mystery is the way red objects in the world are constituted by colourless clouds of molecules [so] reddish experiences merely *inherit* this mysterious air by representing red objects’ (2009, p. 65). The strategy is to ‘...push the mystery allegedly involved in conscious experience out of the mind and into the world’ (2009, p. 65), thereby absolving physicalist positions like SR of any obligation to demystify qualitative character.^9^

I have two objections to this defence. The first objection is that it is very much an open question whether phenomenal qualities are external properties represented by experience, or whether they are instead internal properties of experiences themselves. The external property view is allegedly supported by the so-called transparency of experience (Kriegel 2009, pp. 68–71) but the internal property view looks better equipped to make sense of inversion scenarios.^10^ These, and other, considerations mean that the jury is still out on the nature of phenomenal qualities. If the internal property view is true, then the mysteriousness of qualitative character would clearly be part of the mystery of consciousness. Since Kriegel cannot rule out this view, he cannot assume that the mysteriousness of qualitative character is only an inherited mystery.

The second objection is that even if the external property view is true, this doesn’t necessarily let Kriegel off the hook. A conscious state is the kind of conscious state it is in virtue of its qualitative character i.e. in virtue of its phenomenal qualities. If the external property view is true, then conscious states are typed by the external properties they represent. If those external properties are mysterious, then the properties that type conscious states are mysterious. If the properties that type conscious states are mysterious, then surely demystifying them is a prerequisite of demystifying consciousness. The fact that these qualities have been moved out of the head and into the world doesn’t change the fact that they are integral to the apparent irreducibility of conscious experience. As such, Kriegel would still owe us a demystification of phenomenal qualities. Indeed, other theorists who adopt an externalist view of qualitative character go on to confront the apparent inexplicability of externally-located phenomenal qualities (e.g. Tye 2009). Relatedly, when Kriegel concludes that phenomenal qualities are externally located, he does not infer that they are of no concern for theories of consciousness. Instead, he attempts to incorporate an account of qualitative character into his theory of consciousness. Since Kriegel believes that an explanation of consciousness owes us an explanation of qualitative character, he ought also to believe that a *demystification* of consciousness owes us a demystification of qualitative character.

### Can SR demystify qualitative character?

In the previous section I argued that SR owes us a demystification of qualitative character. The purpose of this section is to determine whether SR can make good on this debt. In Sect. 4.2.1 I look at Kriegel’s positive account of qualitative character. In Sect. 4.2.2 I argue that this account still leaves us with a residual sense of mystery that Kriegel has not dealt with. In Sect. 4.2.3 I consider whether SR can be extended to confront this mystery, but conclude that it is not equipped to do so.

#### Kriegel’s theory of qualitative character

Kriegel arrives at his theory of qualitative character by starting with a familiar simple account of qualitative character. He then identifies a serious problem faced by that account, and shows how that problem can be avoided by introducing his more complex theory. The simple account is that when I look at the sky, my experience has the specific qualitative character it has—say, $\hbox {blue}_{16}$—in virtue of *representing *the sky to have the property of $being\,blue_{16}$. The representation relation itself is then given Dretske’s (1981) teleosemantic analysis: a subject represents $\hbox {blue}_{16}$ just in case they are in a brain state whose function it is to carry information about the presence of $\hbox {blue}_{16}$. Now, this analysis of representation is not without its difficulties. Nevertheless, I think we can grant that this approach, or something quite like it, will ultimately yield a physical reduction of the representation relation (Kriegel 2009, p. 68).

This simple representational account has difficulty accommodating a particular empirical datum regarding colour perception. The datum is that ‘...women apparently perceive surfaces and volumes as ever so slightly darker than how men perceive them’ (Kriegel 2009, p. 84). So when Norma and Norman look up at the sky under the same conditions at the same time and place, ‘...Norma has a visual experience with a $\hbox {bluish}_{17}$ qualitative character, whereas Norman has one with a $\hbox {bluish}_{16 }$ qualitative character’ (Kriegel 2009, p. 85). If the simple representational account is right, then Norma must be *representing* the sky to be $\hbox {blue}_{17 }$ all over, while Norman must be *representing* it to be $\hbox {blue}_{16 }$ all over. Both Norma and Norman’s experiences are veridical: neither of them is victim to a visual illusion. This entails that the sky really is as they each represent it to be. The problem is that ‘...it is impossible for anything to be two different colours all over at the same time’ (Kriegel 2009, p. 85).

This reductio tells us that something has gone wrong, but which step should be rejected? We can’t ignore the empirical evidence in favour of Norma and Norman having different experiences, nor can we bite the bullet and accept that the sky has two contrary properties. One option is to deny that Norma and Norman both have veridical experiences. However, the choice of whose experience is non-veridical looks like it would be arbitrary (Kriegel 2009, p. 86). Furthermore, this account would be committed to saying that half the population consistently has illusory visual experiences, even under ideal conditions. Things are even worse if *both* perceivers are regarded as having non-veridical experiences (Kriegel 2009, p. 86). The more credible option is to reject the simple representational account in favour of a theory that allows both Norma *and *Norman to have veridical experiences. This is what Kriegel tries to achieve with his ‘response-dependent representationalism’.

Let us assume that, rather than representing an occurrent property of the sky, Norma represents the sky’s *dispositional* property of causing certain kinds of perceptual response, in certain kinds of subject, under certain kinds of circumstance. More specifically, let us say that the ‘kind of circumstance’ is normal viewing conditions, that the ‘kind of subject’ is women, and that the ‘kind of perceptual response’ is a neurophysiological state $\hbox {N}_{17}$—specifically, the brain state that Norma and subjects like her go into when they look at a blue sky in normal conditions. Kriegel’s suggestion is that Norma’s experience has a $\hbox {bluish}_{17}$ qualitative character in virtue of representing the sky to have this response-dependent property. Norman’s experience, by contrast, represents a different response-dependent property. He represents the property of being disposed to cause a neurophysiological state $\mathrm{N}_{16 }$ in *male* subjects under normal viewing conditions, where $\hbox {N}_{16}$ is the brain state that Norman and subjects like him go into when they look at a blue sky under normal conditions.^11^

On this view, the difference in the qualitative character of Norma and Norman’s experiences comes with a corresponding difference in content. Importantly though, we can maintain that both experiences are veridical as the sky can have both response-dependent properties simultaneously: it can be both disposed to cause one kind of brain state in one kind of observer *and* disposed to cause a different kind of brain state in a different kind of observer. So although Norma and Norman are having different experiences, neither experience is illusory. By relativizing the content of perceptual experience to the population of the observer, Kriegel’s response-dependent representationalism overcomes the problem faced by the simple representational account. Kriegel is thus led to the conclusion that:

a mental state has the qualitative character it does ... in virtue of representing certain complicated response-dependent properties, characterized in terms of conjunctive dispositions to elicit neurophysiological states in certain subjects under certain conditions. (2009, p. 93)^12^

This account of qualitative character clearly has a number of virtues. The question we must now ask is whether it yields a credible *reduction* of qualitative character, or whether something about qualitative character remains unaccounted for.

#### The residual mysteriousness of qualitative character

Let us grant that the property of representing certain response-dependent properties is amenable to reduction. The question is whether qualitative character is thereby reduced. I would argue that something is left unexplained by this account. Why should representing *that* kind of response-dependent property yield *this *kind of experience rather than some other? When I represent the response-dependent property associated with $\hbox {bluish}_{17 }$ experiences, it appears that my experience could just as easily have had a yellowish qualitative character. There’s nothing about neural state $\hbox {N}_{17}$ that would ‘naturally fit’ a $\hbox {bluish}_{17}$ qualitative character, so we’re left with the mystery of why representing the sky’s property of causing neural state $\hbox {N}_{17}$ should lead subjects to have an experience with that particular qualitative character. In other words, the qualitative character of experience still stands in need of demystification.

One response available to Kriegel is that the quality we are aware of when we have a $\hbox {bluish}_{17}$ experience is one and the same as the relevant response-dependent property. On this view, there is no room for qualitative character to vary independently of the properties our experience represents. There is, however, something odd about saying that the quality $\hbox {blue}_{17}$ is identical with some response-dependent property. Response-dependent properties are dispositional properties. In contrast, phenomenal qualities like $\hbox {blue}_{17}$ appear to be *non-dispositional* properties, where to be a non-dispositional property is to be a property whose nature is not exhausted by any disposition or set of dispositions.^13^ The sky looking blue does not consist in the sky looking to have some power to produce certain kinds of effect. Rather, the sky looks to have the occurrent categorical property of being a certain colour.

Interestingly, Kriegel himself recognises that this presents a serious challenge to his position (2009, p. 94). What he does not recognise, however, is how this challenge parallels the difficulty faced by reductive accounts of subjective character. If you recall, the property of having subjective character resists physical explanation because of its apparent *non-functionalisability*. Now we see that the property of being $\hbox {blue}_{17 }$ generates trouble because of its apparent *non-dispositionality*. But the 2nd-order property of being a non-dispositional property is closely related to the 2nd-order property of being non-functionalisable. Somewhat tautologically, a property is non-dispositional just in case its nature is not exhausted by any dispositional property. Relatedly, a property is non-functionalisable just in case its nature is not exhausted by any *set* of dispositional properties. Just as subjective character appeared to transcend any purely causal characterisation, the qualitative properties of which we are subjectively aware appear to resist any purely causal characterisation too. Kriegel considers two possible responses to the problem at hand: denying that phenomenal qualities appear non-dispositional or accepting this appearance but denying its veridicality. The parallel I have drawn between the apparently disposition-transcending nature of subjective and qualitative character has important consequences for the assessment of both responses.

The first response is the one Kriegel officially advocates, albeit somewhat reluctantly. He claims that ‘...phenomenology itself is simply silent on whether the properties it presents are dispositional or not...’ (2009, p. 95). If $\hbox {blue}_{17}$ doesn’t appear to be non-dispositional, there’s nothing blocking the hypothesis that $\hbox {blue}_{17}$ is a response-dependent property. The difficulty with this move is that denying there is a genuine appearance of non-dispositionality regarding phenomenal qualities is in tension with *accepting* that there is an appearance of non-functionalisability regarding subjective character. In the latter case, Kriegel emphasises the importance of taking appearances of irreducibility seriously and not casting ‘...the philosophic anxiety surrounding consciousness as resting on some kind of confusion’ (2009, p. 267). The very same admonition should encourage him to acknowledge the apparent non-dispositionality of phenomenal qualities. Interestingly, Kriegel does acknowledge that there is something unsatisfactory about denying that phenomenal qualities appear dispositional. He states: ‘I am not entirely comfortable with this answer, because I do think there is a sense in which the phenomenology does not seem to be silent on the issue of dispositionality’ (2009, p. 95). Indeed, there is a wealth of literature suggesting that phenomenal qualities really do appear non-dispositional (e.g. Blackburn 1990; Stoljar 2001; Seager 2006; O’Sullivan 2012). Perhaps, then, the second response is more appropriate.

The second response is to ‘...hold that all conscious experiences represent response-dependent dispositional properties but misrepresent them as response-independent occurrent properties’ (2009, p. 94). However, Kriegel quickly dismisses this response on the grounds that he finds ‘...commitment to such all-encompassing error theory unattractive’ (2009, p. 94). This worry does not sit well with his proposed demystification of subjective character. There, he suggests that subjective character systematically appears to be non-functionalizable when it is in fact (at least according to SR) functionalizable. So why should Kriegel resist positing an analogous error regarding phenomenal qualities?^14^ This response initially looks more promising than the first. After all, Kriegel has already offered a credible account of why subjective character has a misleading appearance, so perhaps the same can be achieved with qualitative character. On Kriegel’s behalf then, I will consider whether SR has the tools with which to demystify qualitative character. If we can explain why phenomenal qualities misleadingly appear non-dispositional, our anti-physicalist intuition will have been neutralised and we will be free to adopt Kriegel’s response-dependent representationalist theory of qualitative character.

How, then, might SR equip us to demystify qualitative character? One interesting possibility is that the very same factor that misleads us as to the nature of subjective character *also* misleads us as to the nature of phenomenal qualities. In other words, Kriegel’s demystification of subjective character might kill two birds with one stone. Although Kriegel does not explicitly adopt this strategy, he does make some comments that encourage such an approach. In a footnote he writes that:

...the mystery concerns in the first instance what makes something a conscious state *in the first place* and derivatively what makes it the conscious state it is *given that it is one.* This suggests that subjective character ought to be the central target of the theory of consciousness, in that the demystification of consciousness would require most centrally the demystification of subjective character. (2009, pp. 11–12 fn. 16)

This passage can be read as saying that qualitative character *does* stand in need of demystification, but that its mysteriousness is parasitic on that of subjective character. As such, a demystification of the latter will *ipso facto* constitute a demystification of the former. After all, Kriegel is explicit about thinking that the mysteriousness of consciousness is likely to have just one source. He recognises the possibility that ‘...several distinct properties suffice individually to generate the mystery in an overdetermining fashion’ but dismisses this possibility as ‘implausible’ (2009, pp. 6–7). If Kriegel deems it implausible that the apparent mysteriousness of qualitative character has an independent source, then he ought to claim that the self-representing nature of consciousness is responsible for this mystery too. Having set the stage, we can now turn to the matter of how exactly the demystification of subjective character could be extended to encompass qualitative character too.

#### Extending the demystification of subjective character

The lesson we learn from the demystification of subjective character is that *if *a mental state suitably self-represents a property it instantiates *then* it will appear to the subject of that mental state that the property instantiated transcends any purely causal characterisation. In other words, it will appear to be a non-dispositional property even if it is in fact dispositional. The quality of which we are aware when we have a bluish experience appears to be non-dispositional. The question, then, is whether the property of which we are aware when we have a bluish experience is a property that our conscious states represent themselves to have. If it *is* a self-represented property, we can import the demystification of subjective character to explain away its apparent non-dispositionality. If it is *not *a self-represented property, then the demystification of subjective character will be unable to account for this appearance, and the apparent irreducibility of qualitative character will persist.

I will give the label ‘C’ to the conscious state in question, which is a perceptual experience of the sky with a bluish qualitative character. I will give the label ‘*P*’ to the hypothetical property of C that meets the following two conditions:

- The Qualitative Character Condition: *P* is identical with the qualitative blueness of C.
- The Self-Representation Condition: *P* is a property that C represents itself to have.

I consider three candidates for *P* but argue that they all fail to satisfy at least one of these two conditions. The three candidates are:

(i) The property of being blue.
(ii) The property of representing [the property of being blue].
(iii) The property of representing {The property of representing [the property of being blue]}.

One might think that a subject in state C is aware of a property in the external world—specifically, the sky’s property of *being blue*. Different theories tell different stories about the nature of this external property: Kriegel, as we have seen, regards it as the response-dependent property of causing certain neural responses in certain subjects under certain conditions. However, this property is a very poor candidate for being property *P*. It is a property of external objects, but qualitative blueness is a property of the conscious state C, so the Qualitative Character Condition is not satisfied. Furthermore, C represents the sky as blue, but does not represent *itself* as blue, so the Self-Representation Condition is not satisfied.

A more sensible suggestion is that *P* is ‘ii’: the property of representing [the property of being blue]. This is a property of C, and one might think that it is the property in virtue of which C has a bluish qualitative character. On the complex account of consciousness offered by Kriegel, C has a lower-order component $\hbox {M}_{1}$ that has the property of representing [the property of being blue]. Moreover, C does represent itself to have this property: C’s higher-order component $\hbox {M}_{2}$ represents this representational property of $\hbox {M}_{1}$, thus C represents its own property of representing [the property of being blue]. This suggests that the Self-Representation Condition is satisfied, but what of the Qualitative Character Condition?^15^ Kriegel holds that the qualitative character of a conscious state is *not* fixed by the first-order representational properties it actually has, but rather by the first-order representational properties that it *represents itself* to have. If $\hbox {M}_{1}$ represented the sky as pink, but $\hbox {M}_{2}$ misrepresented $\hbox {M}_{1}$ as representing the sky as blue, then C would have a bluish qualitative character. Thus, ‘[f]or the state to be qualitatively bluish is for it to be represented to represent the right response-dependent property’ (Kriegel 2009, p. 110). This means that the qualitative character of C is not identical with ‘ii’—the property of representing [the property of being blue]—but rather with ‘iii’—the property of representing {The property of representing [the property of being blue]}. Consequently, the Qualitative Character Condition is not satisfied.

Does ‘iii’ satisfy the conditions of being *P*? As stated, ‘iii’ is identical with C’s bluish quality, so the Qualitative Character Condition is satisfied. The problem is that C does not represent itself to have ‘iii’: C does not have some *third-order *representational property that represents this second-order representational property. As such, the Self-Representation Condition is not satisfied. C represents itself, and C has a particular qualitative character, but C does not represent its own qualitative character. Since the qualitative character of C is not self-represented, the apparently non-dispositional nature of phenomenal qualities cannot be demystified using Kriegel’s strategy.

### Can SR still contribute to the demystification of consciousness?

Although SR fails to demystify qualitative character, its promising demystification of subjective character still stands. So although SR does not provide a *complete* explanation of why consciousness appears irreducible, it does offer a *partial* explanation. Does this mean we should dispense with SR and search for a proposal that offers a complete demystification of consciousness? I suggest that the more sensible option would be to adopt a *divide and conquer* approach to the apparent irreducibility of consciousness. SR neutralises anti-physicalist intuitions regarding subjective character. If it could be combined with some other proposal that neutralises our anti-physicalist intuitions regarding qualitative character, we would have a hybrid account that offers a comprehensive demystification of consciousness.

What would a demystification of qualitative character look like? One possibility is that we misrepresent the nature of phenomenal qualities. Just as the apparent non-functionalisability of subjective character is misleading, so too is the apparent non-dispositionality of phenomenal qualities. I have argued that SR itself is unequipped to explain why we would be victim to such an illusion, but some other account might be offered to explain our situation. Pereboom, for instance, develops the hypothesis that introspection systematically misrepresents the qualities of our experience (Pereboom 2011). If an account could be given of why we misrepresent these dispositional properties as non-dispositional, then we would have precisely what is needed to form a hybrid account. However, no existing theory has succeeded in doing this and I have strong doubts that any future proposals will fare any better.^16^

My preferred route is to acknowledge that phenomenal qualities *really are *non-dispositional properties, and attempt to demystify qualitative character in a manner that accommodates this fact. Specifically, I think that Russellian physicalism has the tools with which to offer such a demystification. As discussed in Sect. 3.2, we generally know entities via their causal dispositions. In fact, physical theory deals exclusively with such properties. A strong case can be made for thinking that these causal dispositions must be grounded in the underlying non-dispositional properties of entities. If our conscious states represent these non-dispositional properties, then we can protect the thought that the properties of which we are aware in experience are non-dispositional.

Of course, a lot more would need to be said about the nature of these non-dispositional properties and about how and why we would represent them in experience. I have attempted to flesh out this proposal elsewhere (McClelland 2013 and the current paper can be read as two halves of a single hybrid explanation of the apparent irreducibility of consciousness). The Russellian physicalist offers a relatively simple demystification of qualitative character. If we attempt to reduce phenomenal qualities to *dispositional *physical properties—specifically, those properties familiar from physical theory—then we will inevitably fail and qualitative character will remain mysterious. If instead we hypothesise that phenomenal qualities are reducible to *non*-dispositional physical properties—specifically, those properties that *ground* the dispositional properties familiar from physical theory—then the sense of mystery will disappear. The apparent conceivability of qualia inverts merely reflects our ignorance of the non-dispositional properties that underwrite the phenomenal character of experience. Although such ignorance prevents us from explaining qualitative character, acknowledging this epistemic blind-spot should alleviate worries that qualitative character is inexplicable in physical terms.^17^

One worry about adopting a divide and conquer strategy is that it appears to be committed to an *overdetermination *of the mystery of consciousness. As discussed in Sect. 4.2.2, one might find it implausible that two separate illusions would conspire to constitute a complex mystery of consciousness. In response I would question whether such overdetermination is really so implausible. The apparent irreducibility of consciousness has often been hailed as one of the hardest problems in metaphysics. The hypothesis that our sense of mystery is overdetermined offers a credible explanation of *why* it so hard: it is so hard because when we attempt to get a grip on the metaphysical status of consciousness there are *two* deep mysteries that impede our progress, so whenever we make headway on one of the mysteries, we are impeded by the other and thus prevented from completing the project. The very *hardness* of the problem of consciousness makes it quite plausible that it is actually an unfortunate amalgam of two metaphysical mysteries. Perhaps a hybrid demystification of consciousness would owe us an account of *why *consciousness should present us with two different puzzles, but I see no reason to doubt that such an obligation could be fulfilled.^18^

In summation, self-representationalism does not have the resources to explain away the apparent irreducibility of consciousness. A genuine demystification of consciousness must address the apparent irreducibility of both subjective character and qualitative character. SR only addresses the former and is not equipped to deal with the latter. Nevertheless, the plausibility of SR’s demystification of subjective character strongly encourages us to pursue a hybrid position that combines SR with some alternative account of the apparent irreducibility of qualitative character.

## Footnotes

1 Kriegel argues that consciousness as a property of *states* has primacy and that consciousness as a property of *creatures* is parasitic on their possession of a conscious state (2009, pp. 23–32). However, those inclined to reverse this order of priority should be able to translate the arguments discussed in this paper into their preferred framework without substantive distortion.

2 There are also two intermediate models according to which subjective character is inseparable from qualitative character but not vice versa, or qualitative character is inseparable from subjective character but not vice versa (Kriegel 2009, p. 53).

3 One could reasonably question whether these scenarios are *genuinely *conceivable rather than merely *appearing *conceivable. However, it is specifically the *apparent *irreducibility of consciousness with which I am concerned here, so this question need not detain us.

4 Issues surrounding the causal inefficacy of non-physical properties generate the most trouble for anti-physicalism (Levine 2003, Chap. 1). Furthermore, when the details of an anti-physicalist theory are fleshed out, the threat of panpsychism looms large.

5 I will assume that this model of reduction is accurate. Kriegel’s demystification is directed at those who believe that consciousness is non-functionalizable, and that its non-functionalizability entails its irreducibility. Either assumption could be called into doubt, but the aim of this paper is to evaluate Kriegel’s proposal on its own terms. It is worth noting that if the anti-physicalists could be defeated even whilst granting them these crucial assumptions, this would be a great victory for physicalism.

6 Kriegel claims that it is crucial that consciousness is an *occurrent *property of mental states rather than merely a *latent *or *potential *property. This is what leads him to reject a ‘functionalist’ theory of self-representation (2009, p. 209). As such, we should regard the causal properties that underwrite suitable self-representation as occurrent causal powers rather than merely latent dispositions. This protects Kriegel’s claim that subjective character is ‘functionalizable’ without collapsing into a ‘functionalist’ theory. The terminology is unhelpful here, but the key point is that Kriegel takes functionalism to entail that having the right *latent* causal powers would suffice for consciousness.

7 It could be argued that subjective character *appearing* non-functionalizable is not our only reason for believing that it *is* non-functionalizable. For instance, Chalmers (1996) takes the conceivability of zombies as evidence that subjective character is non-functionalizable. Here the friend of SR can argue that any putatively independent reasons for regarding subjective character as non-functionalizable actually depend essentially on the initial appearance of non-functionalizability targeted by SR. Thus, in Chalmers’ case, our intuition that zombies are conceivable depends on our initial intuition that consciousness is distinct from its causal profile. So if we have reason to doubt this apparent non-functionalizability then we will also have reason to doubt that zombies are genuinely conceivable. Thanks to Donnchadh O’Conaill for raising this point.

8 Thanks to Donnchadh O’Conaill for highlighting this defence.

9
Byrne (2006) pushes this line and concludes that there is therefore no ‘hard problem’ of consciousness. However, this argument ignores the distinctive explanatory challenge presented by the existence of subjective awareness. Even if colour realism were true, it would be a mystery how physical brain processes could constitute an *experience* of those colours. Of course, it is this sense of mystery that Kriegel seeks to neutralise with his demystification of subjective character.

10 Another point raised in favour of the external property view is that it has good prospects for the naturalisation (i.e. the reduction) of qualitative character (Kriegel 2009, pp. 65–68). This alleged advantage seems spurious to me. If our ultimate goal is to solve metaphysical problems, why would it be advantageous to *relocate* a problem from the mind to the world? Our list of metaphysical problems remains the same length whether we adopt the internal or external property view, so neither view has better prospects for naturalisation than the other.

11 Kriegel refines this account in a number of ways. First, evidence of perceptual differences between subjects of different races and different ages mean that we need to complicate the ‘kind of subject’ component of the response-dependent property. The relevant population might be something like ‘...normal human Asian females in their twenties’ (Kriegel 2009, p. 89). Second, those who (unlike Kriegel) hold that perceptual experiences are multiply realizable will need to complicate the ‘kind of perceptual response’ component. The relevant disjunctive property will be something like $\langle$neural state $\hbox {N}_{17}$*or* silicon state $\hbox {S}_{17 }$*or* ...$\rangle$. Third, as the sky gets darker, Norman will have the bluish$_{17}$ kind of experience that Norma had whilst looking at the sky five minutes earlier. Since having the same qualitative character means representing the same property, the represented response-dependent property had better be conjunctive e.g. ‘...the disposition to elicit a certain response in Norma and her group members in circumstances C *and*a certain response in Norman and his group members in circumstances C*’ (Kriegel 2009, p. 91).

12 We will see in the next section that Kriegel modifies this account of qualitative character. He suggests that qualitative character is not determined by the response-dependent properties we represent but rather by the response-dependent properties that we *represent ourselves *to represent.

13 I stop short of saying that *all *the properties of which we are aware in experience are (or appear to be) non-dispositional. We might, for instance, be able to perceptually experience *affordances*—an external object’s disposition of being usable for certain kinds of action. My claim is that at least some phenomenal qualities appear to be occurrent and, more specifically, that it is occurrent phenomenal qualities that are difficult to accommodate in a representational theory of qualitative character.

14 Thanks to an anonymous referee for drawing my attention to this point.

15 There is a worry here about the *way *in which C represents its property of representing [the property of being blue]. $\hbox {M}_{2}$’s representation of $\hbox {M}_{1}$ is causally mediated. As such, it’s not clear whether we would have the kind of *non-causal *knowledge of $\hbox {M}_{1}$’s properties that Kriegel’s strategy requires. However, since property ‘ii’ fails to satisfy the Qualitative Character Condition, we need not get distracted by this.

16 A critic might object that it is inconsistent of me to accept Kriegel’s error theory regarding the apparent non-functionalisability of subjective character yet reject a parallel error theory regarding the apparent non-dispositionality of phenomenal qualities. In response, I would note that not all error theories are created equal. Kriegel’s arguments make it plausible that we are wrong about subjective character being non-functionalisable. No argument I have seen makes it plausible that we are wrong about phenomenal qualities being non-dispositional.

17 Russellian physicalism accommodates qualitative character by appealing to categorical properties instantiated in the heads of conscious subjects. A comprehensive case for Russellian physicalism would need to rule out views that instead appeal to categorical properties instantiated out there in the world. One possibility is that perceived qualities such as redness are categorical grounds of external objects that ground their dispositions. However, such a view would have difficulty accommodating subjective variation in perceptual experiences of the same object. For instance, if the sky has an objective categorical quality of blueness, how could Norma and Norman’s perceptual experience of the same sky differ in qualitative character? Another possibility is to scrap the appeal to categorical grounds and posit a brute identity between dispositions of external objects and their qualities. However, such a view would still face difficulties with subjective variation and, worse, would renege on the promise of an epistemically transparent explanation of qualitative character.

18 In my (McClelland 2013) I suggest that a hybrid of self-representationalism and Russellian physicalism can tell a relatively simple story about why the two mysteries should coincide.
